# Predictors of intraoperative blood loss in bimaxillary orthognathic surgery: A retrospective multivariate analysis

**DOI:** 10.4317/medoral.28242

**Published:** 2026-06-28

**Authors:** Kazuyuki Yusa, Nobuyuki Sasahara, Tomoharu Hemmi, Kenta Kagami, Kotaro Taniguchi, Shigeo Ishikawa

**Affiliations:** 1 Department of Dentistry, Oral and Maxillofacial-Plastic and Reconstructive Surgery Faculty of Medicine, Yamagata University, Yamagata, Japan

**Keywords:** Blood loss, bimaxillary orthognathic surgery, risk factors, descending palatine artery.

## Abstract

**Background:**

The aim of this study was to investigate the relationship between patient demographics and hematological parameters and blood loss during orthognathic surgery. In addition, we investigated whether bone removal around the descending palatine artery (DPA), a procedure that has been widely applied in recent years, is associated with blood loss.

**Materials and Methods:**

This retrospective study included patients who underwent bimaxillary orthognathic surgery in our hospital. The predictor variables included patient demographics (age, gender and body mass index [BMI]), hematological parameters (hemoglobin level [Hb] and platelet count [PLT]), and surgical factors (bone removal around the DPA). Associations between predictor variables and blood loss were assessed using Spearman rank correlation and the Mann-Whitney U test. Multiple linear regression analysis was performed to identify independent predictors of blood loss during surgery. The level of significance was set as P<0.05.

**Results:**

A total of 138 patients (95 women and 43 men; mean age, 24.1±7.1 years) were included. The mean operative time was 269.1±51.3 minutes, and the mean intraoperative blood loss was 277.0±220.4mL. BMI, preoperative Hb, and operative time were significantly correlated with blood loss (p<.05). Male patients experienced significantly greater blood loss than female patients (p<.05). After adjustment, BMI (B=19.5, p=.001), operative time (B=0.95, P=.010), and removal of bone around the DPA (B=92.8, P=.009) were independently associated with increased intraoperative blood loss.

**Conclusions:**

The findings in this study are important for preoperative risk assessment, surgical planning, and ensuring patient safety in orthognathic surgery.

## Introduction

Orthognathic surgery, including Le Fort I osteotomy (LF1) and bilateral sagittal split osteotomy (BSSO), is one of the most preferred methods for correcting dentofacial problems such as prognathism, retrognathism, maxillary protrusion, maxillary retrusion, open bite and facial asymmetry. These procedures are now widely performed largely due to their safety. However, several complications have been reported. Representative complications include neurosensory disturbances, bad splits, soft tissue and periodontal injuries, fixation material failures, and infections. Numerous studies have examined complications associated with orthognathic surgery and related procedures such as plate removal [1-5]. Although the frequency of these complications has decreased significantly due to various advances, substantial blood loss remains one of the major complications of orthognathic surgery [2]. Some studies have investigated factors affecting blood loss during orthognathic surgery. Notable factors include age, gender, body mass index (BMI), hemoglobin level (Hb), platelet count (PLT), operating time, and surgeon experience [6-8]. Posterior and/or superior repositioning of the maxilla to remove bony interference between the posterior maxilla and pterygoid process is often technically difficult [9]. In recent years, with the widespread use of piezoelectric devices, the resection and removal of bone around the descending palatine artery (DPA) has become commonly practiced in LF1 repositioning [10,11]. However, the degree to which removal of bone around the DPA affects blood loss during orthognathic surgery remains unclear. The aim of this study is to investigate the relationship between patient demographics, hematological parameters, and surgical factors (including the presence or absence of bone removal around the DPA) and blood loss during orthognathic surgery.

## Materials and Methods

*Patients*

For this study, data were collected from 261 patients who had undergone orthognathic surgery in the Department of Dentistry, Oral and Maxillofacial Surgery at Yamagata University Hospital between 2013 and 2023. This retrospective study included patients who underwent bimaxillary orthognathic surgery (LF1 and BSSO) in our hospital. All procedures performed in studies involving human participants were conducted in accordance with the ethical standards of the institutional and national research committee and the Declaration of Helsinki and its later amendments. Approval was obtained from the ethics committee at Yamagata University Faculty of Medicine (approval no.2024-333; date of approval: March 25, 2025). This retrospective observational study was undertaken using the opt-out method of assuming consent via our hospital website.

*Surgery and data analysis*

LF1 and BSSO were performed using standardized methods [12,13]. Depending on the direction of maxillary bone movement (e.g., maxillary impaction and/or posterior reposition), resection and removal of bone around the DPA was performed according to a previous report by Omura *et al.* [10]. All surgeries were performed by experienced surgeons with at least 5 years of experience in orthognathic surgery. Intraoperative blood loss was measured by weighing gauze, measuring suctioned blood, and adjusting for the volume of irrigation solution used during bimaxillary orthognathic surgery. Optimal blood pressure during surgery was maintained by an anesthesiologist in all cases.

This study excluded patients who had bleeding diathesis or connective tissue disease. Cases undergoing either LF1 or BSSO alone or undergoing genioplasty concurrently were excluded. LF1 and BSSO cases other than typical examples (such as cases with distraction or segmental osteotomy) were also excluded.

Information on the following parameters was collected from the medical record: Patient demographics (age, gender and BMI) and hematological indices (Hb and PLT) of patients before surgery and surgical indices (operating time and blood loss during surgery). Blood samples were generally collected within 4 weeks prior to surgery, and BMI was obtained after admission (1-3 days before surgery). Removal of bone around the DPA during LF1 was also confirmed from the medical record of patients. Patients with incomplete data were excluded.

*Statistical analysis*

All statistical analyses were performed using Jeffreys’s Amazing Statistics Program (JASP, University of Amsterdam, Amsterdam, the Netherlands). Correlations between variables were assessed using the non-parametric Spearman’s rank correlation technique. The Mann-Whitney U test was used to examine the relationship between blood loss during surgery and gender, as well as presence or absence of removal of bone around the DPA. In addition, the associations of age, BMI, Hb, PLT, operating time, gender and removal of bone around the DPA with blood loss during surgery were evaluated using multiple linear regression. Age, BMI, Hb, PLT and operating time were treated as continuous variables, while gender and removal of bone around the DPA were treated as categorical variables. The level of significance was set as *p<*0.05.

## Results

A total of 138 patients (95 women, 43 men; mean age, 24.1±7.1 years) were included in this study (Figure 1). Mean BMI was 21.6±3.1kg/m^2^. Mean Hb was 13.8±3.1g/dL and mean PLT was 257.7±51.3×10^4^/μL. Mean operating time was 269.1±51.3min and mean blood loss was 277.0±220.4mL. Removal of bone around the DPA during LF1 was performed in 78 cases (Table 1). Table 2 shows the correlations between variables (age, BMI, Hb, PLT and operating time) and blood loss during the surgery. BMI, Hb and operating time correlated with blood loss during surgery (BMI: rs=0.279, *P<*0.001; Hb: rs=0.265, *P=*0.02; operating time: rs=0.311, *P<*0.001). In addition, the Mann-Whitney U test revealed significantly greater blood loss during surgery in males (*P<*0.05). Although a tendency toward higher blood loss during surgery was observed in the group with removal of bone around the DPA, the difference was not significant (*P=*0.133) (Figure 2). Further, Table 3 shows the results of the relationship between each variable and blood loss during surgery using multiple linear regression. The effect of each variable was estimated after adjusting for the other variables, resulting in BMI (B=19.517, *P=*0.001), operating time (B=0.950, *P=*0.010) and removal of bone around the DPA (B=92.823, *P=*0.009) being identified as influences on blood loss during surgery.

**Discussion** 

The present study aimed to identify factors associated with blood loss during bimaxillary orthognathic surgery. Consistent with previous reports, the effects of patient demographics, hematological indices such as Hb and PLT, and surgical variables such as operating time on intraoperative blood loss were examined. In addition, the presence or absence of bone removal around the DPA during LF1 was investigated as an approach that has become increasingly common in recent years. Given recent advances in orthognathic surgery, including expanding indications and patient preferences, it is anticipated that the number of cases involving bone removal around the DPA will continue to increase, and the significance of this study is likely to grow [14,15]. Regarding patient demographics, male patients tended to experience greater blood loss during surgery (Figure 2), in accordance with previous reports [16-18]. Olsen *et al*. [19] similarly reported higher blood loss in males and noted that this trend is also observed in procedures such as joint arthroplasty and hepatectomy [20-22], suggesting that this finding may be attributable to gender differences in fibrin turnover.

In the present study, a positive correlation was observed between BMI and intraoperative blood loss (Tables 2 and 3). In orthopedic surgery, several reports have indicated that increased BMI can influence intraoperative bleeding [23,24].

In orthognathic surgery, some studies have found no association between BMI and blood loss [25], whereas others have suggested that higher BMI, particularly obesity, is associated with increased blood loss [8,26]. Although previous results are inconsistent, elevated BMI should be considered a potential risk factor for increased blood loss during bimaxillary surgery. A correlation was also observed between Hb and intraoperative blood loss (Table 2). Topan *et al*. similarly reported that higher Hb levels were associated with increased intraoperative bleeding, and that higher preoperative PLT and mean platelet volume/PLT values corresponded to reduced overall blood loss following orthognathic surgery [8]. Although this study found no association between intraoperative blood loss and PLT (Tables 2,3), overall blood loss was not assessed, and hematological values may vary depending on the timing of blood sampling. The relationship between hematological parameters and surgical blood loss thus remains unclear and warrants further investigation. Prolonged operating time was also associated with increased intraoperative blood loss, consistent with numerous previous reports [8,16,26-28]. In addition to these factors, this study evaluated the influence of bone removal around the DPA and found that its presence was associated with  a slight increase in intraoperative blood loss (Table 3).

With regard to additional procedures and bleeding in orthognathic surgery, Kretschmer *et al*. reported that additional osteotomies and iliac crest grafts contributed to increased blood loss [16]. Although removal of bone around the DPA is a highly useful and common technique, no prior studies have examined its effect on blood loss. The present findings suggest  that this procedure may slightly increase bleeding;  however, the amount was not clinically significant enough to discourage its use. With appropriate bleeding control measures, bone removal around the DPA can be performed safely when indicated.

Understanding the potential risks of blood loss during bimaxillary surgery, as described above, is crucial for providing safe surgical care. Depending on the situation, measures such as hypotensive anesthesia or combined medication use may help reduce blood loss. Further, preparation for interventions that may reduce blood loss, such as hypotensive anesthesia [29] and combined medication use [30,31] may also be important.

This study had several limitations. First, a retrospective design was used. Because the purpose was to obtain preliminary findings, no statistical power analysis was conducted, and the sample size was determined by the number of eligible patients during the study period. A prospective study with an appropriately calculated sample size will be required in the future. Furthermore, these results should be interpreted with an understanding  of the study’s limitations. Second, the method of measuring blood loss was an issue. Blood loss is most commonly calculated by measuring suctioned blood and adjusting for the volume of irrigation solution, with the addition of data about the weight of surgical gauze [28]. However, this approach does not fully account for blood redistribution into the third space. Future research may need to incorporate assessment of total blood loss, rather than intraoperative loss alone. Third, multiple surgeons were involved in the procedures analyzed in this study. Rummasak *et al*. reported a correlation between surgeon experience and intraoperative bleeding [18], whereas Kretschmer *et al*. found no significant difference between resident and experienced surgeons [32]. In the present study, all surgeries were performed by surgeons with over 5 years of experience in treating jaw deformities, indicating a uniformly high level of technical proficiency. However, further studies minimizing surgeon-related bias are warranted. Additionally, the exclusion of factors such as intraoperative anesthesia status and the direction of maxillary bone movement from the analysis may also be considered a limitation.

## Conclusions

Despite these limitations, this study may provide useful insight into factors influencing blood loss in bimaxillary surgery, including the impact of bone removal around the DPA. These findings may be helpful for preoperative risk assessment, surgical planning, and ensuring patient safety in orthognathic surgery.

## Figures and Tables

**Table 1:** Table 1: Characteristics of the 138 patients.

**Age (years)**	
*Mean±SD*	24.1±7.1
**Gender**	
*Male; n (%)*	43 (31.2)
*Female; n (%)*	95 (68.8)
**BMI (kg/m** ^2^ **)**	
*Mean±SD*	21.6±3.1
**Hb (g/dL)**	
*Mean±SD*	13.8±1.6
PLT (×10^4^/mL)	
*Mean±SD*	257.7±51.3
**Operating time (min)**	
*Mean±SD*	269.1±46.6
**Blood loss (mL)**	
*Mean±SD*	277.0±220.4
**Removal of bone around the DPA**	
*+; n (%)*	78 (56.5)
*-; n (%)*	60 (43.5)

**Table 2:** Table 2: Correlation between blood loss during surgery and age, BMI, Hb, PLT and operating time.

**Variables**	**rs**	**P**
Age	-0.013	0.879
BMI	0.279	<0.001***
Hb	0.265	0.02*
PLT	0.053	0.535
Operating time	0.311	<0.001***

**Table 3:** Table 3: Multiple linear regression analysis of factors associated with blood loss.

**Variables**	**B**	**SE**	**β**	*P*
Age	-2.76	2.383	-0.088	0.249
BMI	19.52	19.578	0.277	0.001**
Hb	26.88	15.702	0.190	0.089
PLT	0.100	0.330	0.023	0.762
Operating time	0.950	0.363	0.201	0.010*
Gender (M/F)	51.358	56.141		0.362
Removal of bone around the DPA	92.823	34.738		0.009**

Statistically significant: *p<0.05; **p<0.01.

**Figure 1:**
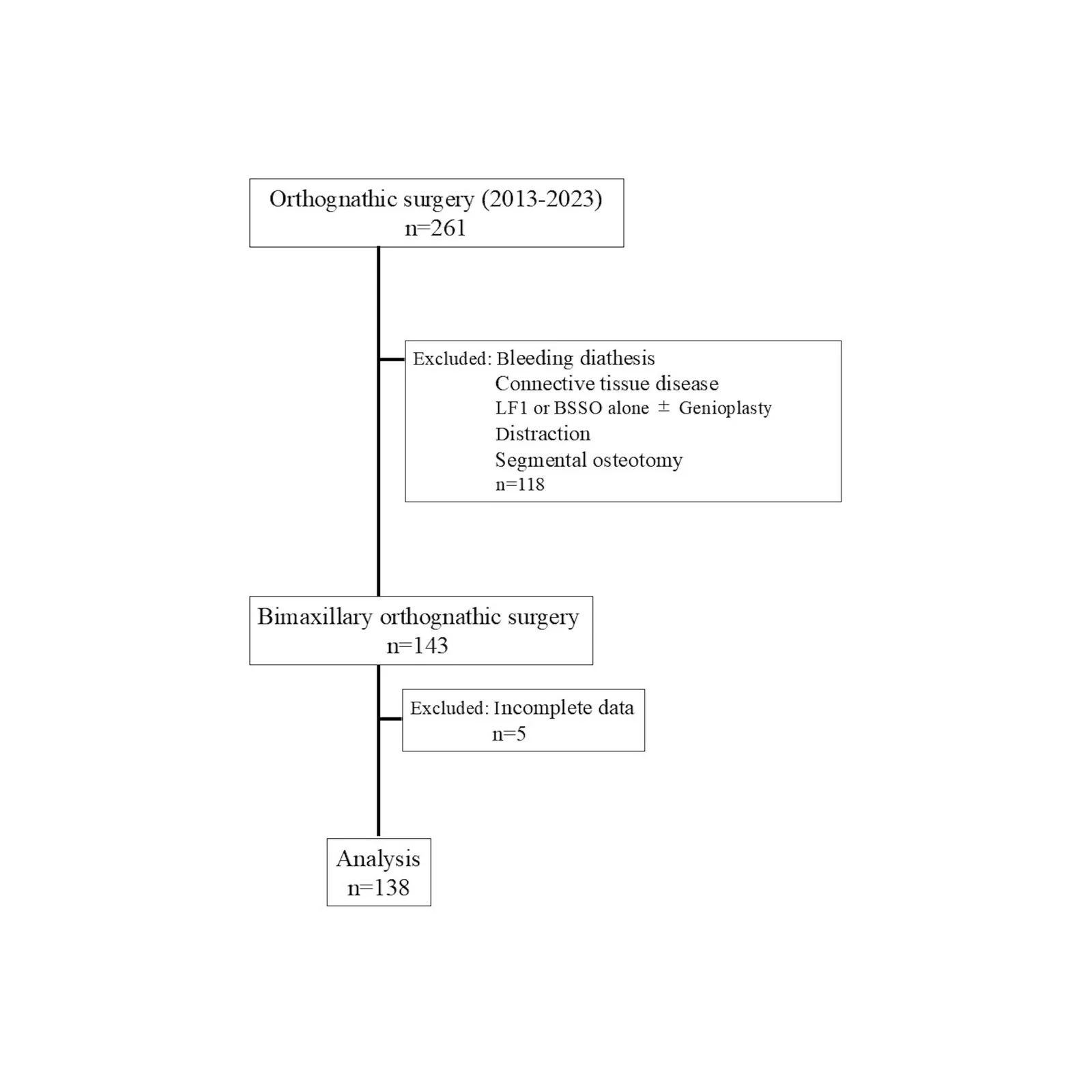
Fig. 1: Flowchart for the selection of cases in this study.

**Figure 2:**
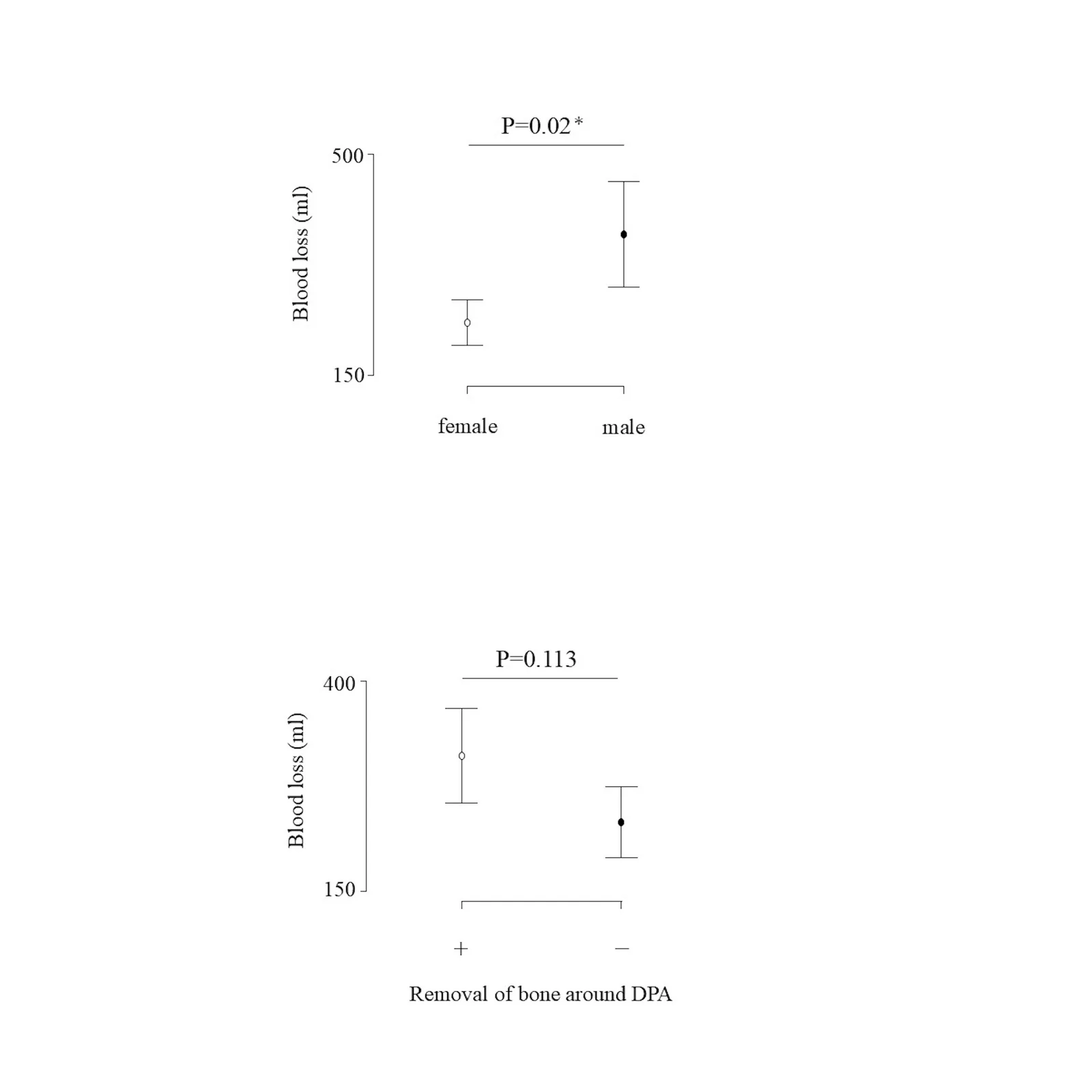
Fig. 2: Blood loss in female and male patients (A) and presence or absence of removal of bone around the DPA (B). The median blood loss was 328 (185-510) mL in males and 200 (100-368) mL in females; males had significantly greater blood loss (P=0.02) (A). Furthermore, although the median blood loss in the group with removal of bone around the DPA was 253 (127-450) mL, which was higher than that in the group without bone removal around the DPA (212 (112-344) ml), the difference was not statistically significant (P=0.133) (B). *p<0.05.

## Data Availability

None.
